# ‘It takes a village’: patient lived experiences of residential treatment for an eating disorder

**DOI:** 10.1192/bjo.2024.849

**Published:** 2025-02-03

**Authors:** Rebekah Rankin, Janet Conti, Lucie Ramjan, Phillipa Hay

**Affiliations:** Translational Health Research Institute, School of Medicine, Western Sydney University, Sydney, Australia; School of Psychology, Western Sydney University, Sydney, Australia; School of Nursing and Midwifery, Western Sydney University, Sydney, Australia; Mental Health Services, Campbelltown Hospital, South Western Sydney Local Health District (SWSLHD), Sydney, Australia

**Keywords:** Anorexia nervosa, feeding or eating disorders, lived experience, qualitative research, residential treatment

## Abstract

**Background:**

Residential treatment facilities for eating disorders are becoming increasingly common and purport to provide recovery-orientated care in a less restrictive environment than traditional hospital settings. However, minimal attention has focused on individuals’ lived experiences of these residential services.

**Aims:**

This study explores participants’ lived experiences of care at Australia’s first residential facility for the treatment of eating disorders.

**Method:**

Qualitative data were collected as part of a clinical evaluation (June 2021 to August 2023). Fifteen women participated in semi-structured interviews about their experience of treatment following discharge. Data were analysed with inductive reflexive thematic analysis.

**Results:**

Three main themes were generated from the data that included participants’ journeys to treatment, experiences of treatment and the transitions associated with and following discharge. Cutting across these main themes were participants’ encounters of barriers, setbacks and hope. Participant experiences of residential treatment were complex and multifaceted, marked by inherent ideological dilemmas that arose in balancing standardised treatment protocols with person-centred and recovery-oriented care. Participants also spoke of reclaiming a sense of self and identity beyond their eating disorder, emphasising the importance of relationships and consistent and collaborative care.

**Conclusions:**

Participant accounts of residential treatment emphasised the importance of holistic, person-centred and recovery-oriented care. Despite the complexities of treatment experiences, participant narratives underscored how recovery may be more about the reclamation of a sense of identity outside of the eating disorder than merely symptom improvement. As such, adopting person-centred and recovery-oriented treatment approaches within residential treatment settings may maximise individual autonomy and promote holistic recovery pathways.

Eating disorders are complex and potentially life-threatening psychiatric illnesses, characterised by disturbances in eating or eating-related behaviour leading to impairments in physical health and/or psychosocial functioning.^1,2^ Globally, the lifetime prevalence rate for formally diagnosed eating disorders is estimated to be 8.4% (range 3.3–18.6%) for women and 2.2% (range 0.8–6.5%) for men.^3^ Treatment for eating disorders is typically provided along a continuum of care, often commencing with regular out-patient consultations involving psychologists, primary healthcare providers and/or dietitians.^4^ Instances of medical instability, suicidality or low treatment engagement may necessitate more intensive interventions, such as intensive out-patient treatment or in-patient hospital admission. Despite the development of evidence-based interventions, such as enhanced cognitive–behavioural therapy^5^ and family-based therapy,^6^ the course of eating disorders is commonly protracted with high rates of remission.^7^ Long-term follow-up studies indicate that 30–64% of individuals who received in-patient treatment for an eating disorder still met diagnostic criteria 10–20 years following treatment.^8,9^

Current evidence indicates that, without improvements in both physiological and psychological aspects of an eating disorder, there exists a risk of a pseudo-recovery – that is, physical recovery in the absence of psychological recovery – and increased risk of remission.^10^ Thus, although medical safety is essential in ameliorating both psychological and psychosocial symptoms of an eating disorder,^11,12^ individual aspects are important to consider in predicting treatment adherence and patient outcomes.^13^ For example, stigma, shame and guilt are frequently identified as the most impactful barriers to individual motivation and subsequent treatment engagement, alongside practical barriers such cost of treatment and location.^14,15^ Furthermore, although many individuals with longstanding eating disorders are labelled as ‘treatment resistant’,^16^ it is also possible these individuals may have been unable to access treatment tailored to their needs and preferences.^17^

## Lived experiences of treatment

The gap between in-patient and out-patient treatment for eating disorders is substantial.^7,8,14^ In-patient treatment programmes have traditionally centred on models of refeeding and medical stabilisation and weight restoration, employing a ‘one-size-fits-all’ approach to care. A recent review^18^ exploring patients’ lived experiences of in-patient treatment for anorexia nervosa found that, although many individuals recognise the necessity of medical intervention, they often express feeling marginalised by the restrictive treatment environment and biomedical focus of in-patient treatment facilities.^19^ Conversely, day- and out-patient treatment offers greater flexibility, but frequently lacks the resources and intensity needed to support recovery. This disparity underscores the need for integrated care models that facilitate continuity of support in this transition.^11,14,17^ The review^18^ also highlighted the multifaceted nature of treatment experiences, and acknowledged the ‘inherent conflicts’ in balancing the necessity of medical and psychological intervention with person-centred approaches.

## Residential treatment for eating disorders

Residential treatment programmes for eating disorders have gained popularity in recent years, expanding from the USA to countries such as Canada, Italy and Australia as well as the UK.^20^ These programmes offer intensive 24/7 treatment in a ‘home-like’ environment and are designed to support individual recovery over several weeks to months. A critique highlighted in the literature regarding in-patient and intensive treatment facilities is the presence of a dominant biomedical discourse that often disqualifies the voice and identity of the individual seeking treatment.^21,22^ Residential treatment programmes typically place a stronger emphasis on fostering individual autonomy and psychological recovery than in-patient settings and, as such, are intended for individuals who are medically stable, but require a higher level of treatment intensity than what may be offered in the out-patient or day programme settings.^23^ Treatment in the residential setting is commonly provided by a multidisciplinary team and involves regular meal support, low-intensity medical monitoring, and individual and group psychotherapy using traditional evidence-based approaches such as cognitive–behavioural therapy, as well as adjunctive therapies such as yoga.^23,24^

A systematic review of 19 open-label trials^23^ and a large (*n =* 1421) retrospective clinical outcomes trial reported positive outcomes associated with residential-based treatments. A more recent scoping-review (*n =* 12) of outcomes associated with residential programmes for eating disorders found moderate to strong evidence to support positive outcomes for patients, such as eating disorder psychopathology, weight restoration, quality of life, anxiety, depression and cognitive functioning.^24^ Despite the growing number of residential programmes and evidence suggesting that residential facilities may be an effective modality of eating disorder treatment, there is a paucity of published literature exploring participants’ lived experiences of residential treatment for an eating disorder.

## Study aims

Given the limited clinical evaluation in the literature, it is unclear if the aspirations of the residential model are meeting the perceived needs of those seeking treatment. As such, understanding participant lived experiences is crucial for the ongoing development of this model of care and improving treatment outcomes. This study aimed to explore the participant lived experiences of care at Australia’s first residential facility for the treatment of eating disorders.

## Method

### Ethics

The current study is a component of a wider clinical evaluation of the first residential treatment facility for the treatment of eating disorders in Australia. This study was registered with the Australian New Zealand Clinical Trials Registry (identifier ANZCTR12621001651875p). The authors assert that all procedures contributing to this work comply with the ethical standards of the relevant national and institutional committees on human experimentation and with the Helsinki Declaration of 1975, as revised in 2013. All procedures involving human patients were approved by the Western Sydney University Human Research Ethics Committee (approval number H14742). All participants provided written and verbal informed consent.

### Facility

Participants were recruited from a single residential facility for the treatment of eating disorders in Australia. The purpose-built facility is staffed by a multidisciplinary team who provide 24/7 residential care and support for up to 13 individuals in a home-like environment. The facility employs the Butterfly Foundation Residential Eating Disorder Treatment (BFREEDT) Model of Care, which is a six-phase treatment structure based on the work of Carolyn Costin^25^ and underpinned by the principles of best practice outlined in The Royal Australian and New Zealand College of Psychiatrists clinical practice guidelines for the treatment of eating disorders.^26^ Full admission criterion for the facility are outlined in Supplementary File 1.

### Recruitment

All (*n* = 84) patients admitted for treatment during a 25-month period (July 2021 to August 2023) were invited to participate in a clinical evaluation of the residential facility. Consenting participants were contacted by the first author, following discharge, and invited to participate in an interview about their experiences of residential treatment. This was done to ensure equitable participation opportunities.

**Fig. 1:**
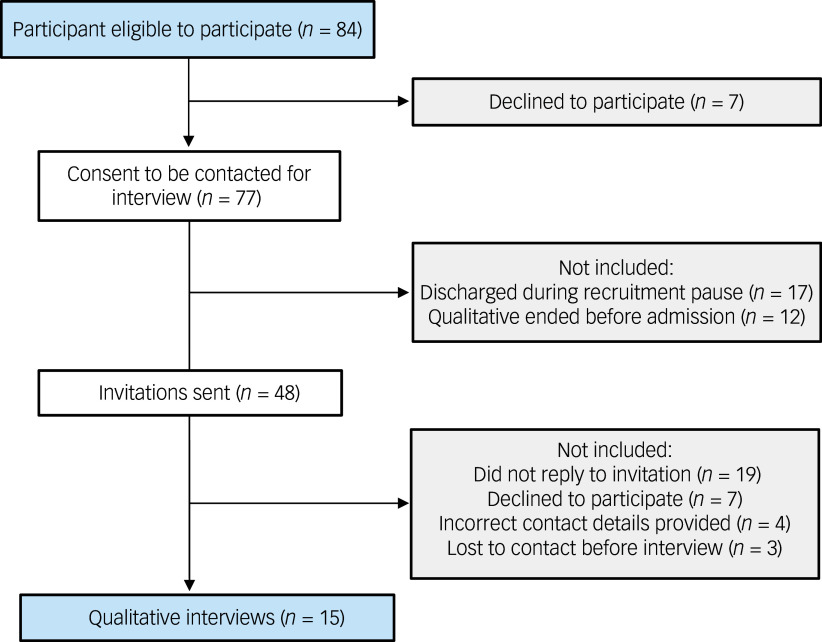
Flow of participant recruitment.

For pragmatic purposes, the authors anticipated a sample size of approximately 10% of admissions would be sufficient to generate adequate data to depict a nuanced and multifaceted picture of the patterns pertaining to participants’ lived experience of residential treatment.^27^ Once this number was reached, recruitment continued in a reflexive manner until the researchers were confident there were multiple representations of a range of possible participant experiences – positive, mixed and negative – resulting in a sample of 17.86% of the total admissions. Recruitment and participation rates are shown in Fig. 1.

### Participants

Fifteen women (mean age 27.9 years, s.d. = 5.12) consented to participate in an interview about their residential treatment experience. The majority (*n* = 12) of participants identified as being Australian of Anglo European descent. Mean illness duration was 11.5 years from diagnosis (s.d. = 4.26; range 4–19 years) and mean length of admission was 74.89 days (*n* = 17; s.d. = 48.52 days). Approximately two-thirds of those interviewed (*n* = 9) described themselves as being actively engaged in recovery, with the remaining participants describing themselves as having experienced a setback or considering in-patient admission because of relapse. Two-thirds of participants were diagnosed with anorexia nervosa (majority 80% restrictive subtype) and a third with atypical anorexia nervosa. All participants reported multiple prior in-patient admissions for treatment related to their eating disorder diagnosis.

### Data collection

Data were collected through a semi-structured interview following discharge from the facility. This approach was employed to safeguard participant confidentiality because of the limited number of eligible individuals, and to foster an environment conducive to open, reflective sharing of experiences, including challenges faced in the facility or milieu. The interview was purposefully designed by the authors in collaboration with a lived experience advisory group (see Supplementary File 2). The interview schedule was informed by a narrative therapy framework^28^ as outlined in the Experience and Identity Interview.^29^ This framework allowed participants to raise experiences, meanings and self-reflections that were pertinent to their lived experience of the service through a process of self-narration. Clinical diagnostic data were obtained from the electronic medical record.

All interviews were conducted by the first author, a clinical psychologist and researcher external to the facility, and lasted approximately 60 min (mean 60.47 min; s.d. = 2.25). To accommodate participant locations and scheduling preferences, interviews were conducted via Zoom (version 5.10.6–5.15.13 for Mac iOS, Zoom Communications, Denver, CO, USA; https://www.zoom.com) and audio recorded. Following the interview, participants were debriefed by the first author and reimbursed $50 for their participation.

### Analysis

Interview recordings were transcribed and de-identified with participant-chosen pseudonyms. Further identifying information was removed (e.g. locations, dates, affiliations) following participants’ review of their interview transcript. De-identified transcripts were exported to NVivo (version 14 for Mac iOS, Lumivero, Denver, CO, USA; https://www.lumivero.com) and analysed with inductive reflexive thematic analysis within a social constructionist framework.^30^ This approach assumes a bidirectional relationship between language and experience, viewing language as integral to the social (re)production of participants’ meaning and experiences. The authors’ choice of a narrative therapy informed interview schedule was consistent with the reflexive analytic approach.

Transcripts were coded and a set of themes and subthemes developed (by R.R.) according to Braun and Clarke’s^27^ six-phase approach to thematic analysis and reported in line with the Reflexive Thematic Analysis Reporting Guidelines.^31^ Because of the exploratory nature of the research, an inductive coding approach was adopted that was driven by participant experiences rather than influenced by pre-existing theories or researcher preconceptions. Coding notes underwent collaborative review for overlapping and repetitive codes by the authors (R.R., J.C., L.R., P.H.) before being consolidated into themes. Similar themes were grouped together to identify major patterns in the data, thus forming overarching themes and subthemes. Extracts that most accurately represented the final set of themes (see Table 1) were incorporated into the analytical narrative to create a meaningful representation of participants’ experiences.

**Table 1:** Participant extracts^a^

Theme	Subtheme	Exemplar quote (participant pseudonym)
1. Journeys to treatment	a. A loss of identity	I was too unwell and was hospitalized quite a fair bit… physically I was compromised [diagnostic details necessitating medical intervention]… It becomes challenging to juggle life as well as an eating disorder. There kind of comes a point where something’s got to give, I guess, and to be honest it’s usually not the eating disorder. (April) I think it [the eating disorder] was helping me in a sense of managing emotions and trauma that was coming up for me. So it was helpful in that sense, but I did lose a lot of my identity to the anorexia. (Lily) … it [the eating disorder] was how I survived life. (Sam)
	b. A ‘Hail Mary’	… it’s kind of like a Hail Mary. It’s like a last ditch. You feel like there is no life after this. It seems that you’ve been in this so long that you can’t imagine your life without it [the eating disorder]. (Mia) I kind of went to [residential facility] as like kind of the last resort thing, because I had exhausted all of my options and I also like had gotten a lot of medical trauma from hospital and all that as well. (Jessica) It kind of got to a point in my illness…, where I had just had enough. I was exhausted… what other hope did I have? If nothing else had worked, this was something that I hadn’t tried. (Hannah)
2. Experiences of residential treatment	a. Giving me an identity	I think it was the personalised approach and it was seeing me as [Enola] and not “a girl with anorexia”. It was, you know, looking… talking to me about things other than my eating disorder. Doing things other than the eating disorder. … Giving me an identity. (Enola) It was just liberating to be not a patient but a person. … Being able to do normal everyday things and practice those things… I expected it to be like hospital, but it wasn’t, it was more like a home… (Hannah) … the fact that, like even 2 days after you got there, you got to go out for breakfast – that was wild. (Abigail) Definitely the collaborative non-punitive approach was vastly different than anything else and really made a difference, it encouraged me to reflect on why I was there for myself. (Lily)
	b. Consistency and communication	I have to say from my personal journey it was… I walked into every MDT [multidisciplinary team] meeting, essentially to a room full of cheerleaders. … but I just know from others if they had had a week where potentially they hadn’t met certain goals and… they’d stalled a little bit. They would definitely come out [of the MDT meeting] feeling like they’d had a slap on the wrist. (Sarah) … something that I needed without realising, was the being away from just everything to focus solely on recovery. (Hannah)
	c. Recovery takes a village	I think, eating disorders are not a thing that you can heal alone. … Like, “it takes like it takes a small village to raise a child.” I think it’s much the same with an eating disorder. It takes a small village and a community to like heal the person and to build up that trust to move forward. … you can’t do it by yourself. You can’t work it out alone. You can’t do it without people around you… it just doesn’t work. (Mia) … There’s a lot of people [staff] here who’ve had eating disorders, and I look at them now I think they’re incredible. They are all in different bodies. They have it all - different jobs and life stories. … Like, they’re killing it. Like, I would be so happy if my life looked like theirs. (Jenna) While Wandi isn’t perfect, it can be life changing. And it… a lot of that comes from the people involved in Wandi. (Michelle) … there were other people who were further along in their stay there who were really committed to recovery, and you could see that and hear that. That had a really positive impact. (Abigail)
3. Transitions		I think the discharge planning is probably not working as thoroughly or as well as it should be. … So I think more thought for discharge planning is needed. Because there is no step down into the community. And integrating that with other health services to sort of ensure that it’s cohesive. (Megan) … your recovery journey doesn’t end with [facility name]. … you kind of go through it [the programme] and it gives you the tools to recover for sure, but it stops and cuts off… and the journey doesn’t end there all the time – it keeps going. (Mia) I think it’s really, it is a very difficult transition… [following discharge] it really did feel like you were just dropped. (Grace) When I go to public places and I’m [often] under the Mental Health Act. … Not having the freedom of choice to leave when I want to or have any kind of freedom is quite scary and it doesn’t allow you to keep going. Whereas if I’m doing it on my terms and I’m doing it because I want to get better…, that allowed me to keep going [at Wandi]. … I didn’t feel trapped. (Enola)
Cross-cutting theme	Barriers, setbacks and hope	I just ended up not really embracing it fully… I didn’t feel like I was involved in the goals setting or like I didn’t feel like I had any say in my treatment. But I did feel that the staff were really caring and compassionate, and I did feel like it was a healing environment. (Grace) Like it was my choice to come to [treatment] to begin with, but then it kind of got hijacked by my [family member… [they] really took control of the whole situation. … this thing that I had chosen to do, then became not my choice. (Bourke) There’s such a monetary involvement with that programme that it’s almost excluding people in the middle class from receiving health care. … (Sage)

a. All names are participant-chosen pseudonyms.

### Positionality

In adopting reflexive thematic analysis within a social constructionist perspective, the authors acknowledge the influence of researcher positionality in the processes of data generation, analysis and presentation. Detailed author positioning statements can be found in Supplementary File 3.

## Results

**Fig. 2:**
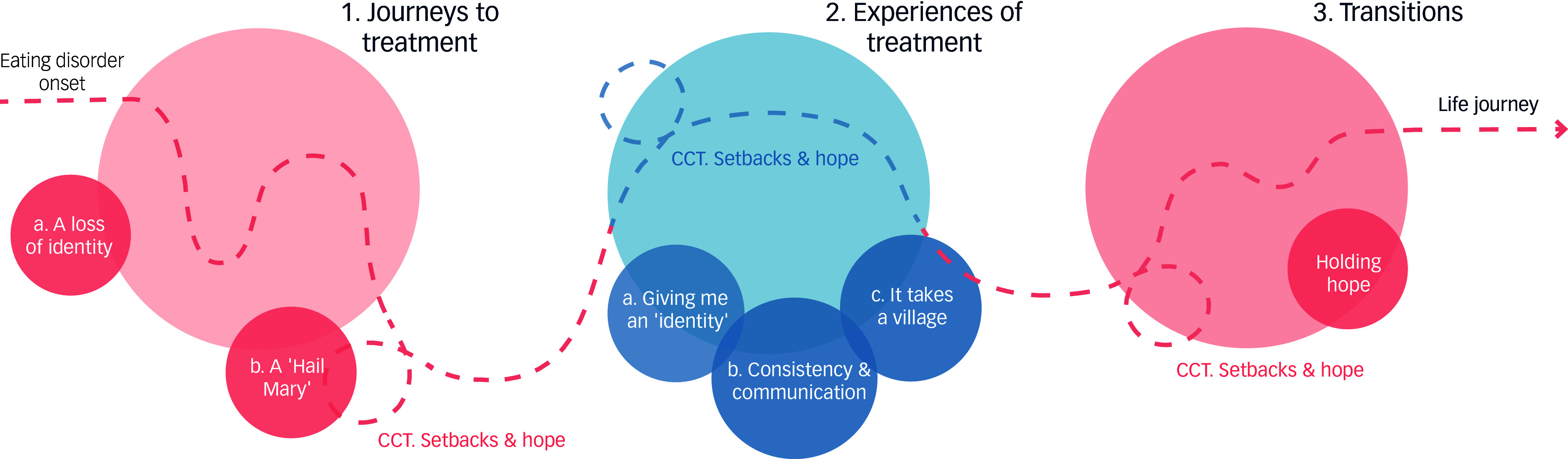
Thematic map of participant experiences. CCT, cross-cutting theme.

In exploring participants’ lived experiences of treatment at Australia’s first residential treatment facility for eating disorders, three themes and one cross-cutting theme were generated from the data. As depicted in Fig. 2, these themes are explored in narrative sequence following participants’ *journeys to treatment* (theme 1), *experiences of residential treatment* (theme 2) and the *transitions* associated with and following discharge (theme 3). Throughout their journey, participants encountered *barriers, setbacks and hopes* (cross-cutting theme 1). Exemplar participant extracts are presented in Table 1.

### Theme 1: journeys to treatment

Participants’ journeys to treatment were unique and varied. All participants reported complex and extensive treatment histories along the continuum of care, spanning several years to decades. Participant treatment histories were punctuated by in-patient treatment and experiences of restrictive practice, encapsulating a cycle akin to a ‘revolving door’ (Daphanie) of steps toward recovery followed by episodes of medical instability. Inherent within participant narratives was an acknowledgment of the necessity of medical in-patient interventions as part of their treatment journey. However, the treatment environment and biomedical focus of these facilities were often experienced as restrictive, leading to the disqualification of participants’ voices, individual identities, lived experiences, personal values and understandings of eating disorder symptoms. Thus, participants described a loss of identity along their treatment journeys and saw residential treatment as being a ‘Hail Mary’ (Mia).

#### Theme 1a: a loss of ‘identity’

When asked to share the story of their eating disorder, participants frequently reflected on the pervasive influence the eating disorder had on their lives. Participants frequently spoke of the perceived totality of the eating disorder, particularly when speaking to their treatment histories. Within these treatment histories, participants described a complex interplay between the disorder and their sense of self. Individual narratives painted a picture of complex and recursive identity negotiations as they sought to navigate a life that had become dominated by the eating disorder. In speaking on the impact of the eating disorder on their time, relationships, hobbies and careers, participants frequently reflected on how their identity became lost to the eating disorder identity. This sentiment was effectively encapsulated in Lily’s reflection: ‘… I did lose a lot of my identity to the anorexia’. Despite recognising the detrimental effects of the eating disorders, all participants outlined the struggle to imagine or contemplate the concept of a life without the eating disorder. These discussions highlighted participants’ perceived absence of personal agency to act on their own behalf in the face of their eating disorder and its effects on their life.

#### Theme 1b: a ‘Hail Mary’

Paralleling the participants’ experiences of the dominance of the eating disorder were narratives of resistance, for example having ‘had enough’ (Hannah) of the eating disorder and its increasingly untenable effects. Within the broader context of recursive treatment attempts, participants described looking for something ‘different’ from the continuum of treatment options available to them. Many individuals described having followed the development of the residential facility since its inception, several years before their admission, in the hopes that they would not need to access the treatment service. Within the broader context of their treatment journeys, participants viewed residential treatment as being like a ‘Hail Mary’ or ‘last ditch’ (Daphanie) attempt at holding on to hope for recovery and finding a life beyond the eating disorder.

### Theme 2: experiences of residential treatment

As with their journeys to treatment, participants’ experiences of residential treatment for an eating disorder were unique and varied. In reflecting on their treatment experiences, participants rarely commented on a specific treatment modality, evidence-based intervention or behavioural changes. Rather, they almost exclusively reflected on themes of self, identity, connection and hoped-for futures.

#### Theme 2a: ‘giving me an identity’

A central tenet of the service’s treatment philosophy was the concept that each individual embodies both a ‘healthy self’ and an ‘eating disorder self’. As such, pivotal objectives of the programme enhanced participant recognition of these selves, with the aim of acknowledging the function of the eating disorder self and strengthening the healthy self.^25^ When asked to reflect on their treatment experiences, participants rarely reflected on the language of healthy self or eating disorder self, but rather reflected on treatment as ‘giving me an identity’ (Enola). As such, individual experiences of treatment appeared to be less about the illness process and more about the reclamation of self and identity outside of the eating disorder.

Participants also spoke of treatment as being ‘collaborative’ and ‘non-punitive’. This approach, in combination with consistent and compassionately supported meal exposure, was experienced as helpful, particularly in the context of previous treatment experiences that were perceived as being ‘restrictive’ and ‘punitive’. Participants reflected that the individualised approach was empowering and had them feeling seen a person rather than ‘[just] a girl with anorexia’ (Enola).

Participants also frequently reflected on the value of being afforded the dignity of normality. For example, when participants spoke of the treatment environment, they frequently spoke of the value of normality in the small things such as being able to wear ‘normal clothes’ (Enola), the casual dress of staff, food being presented on ‘normal plates’ and in an aesthetically pleasing manner, being able to access the outdoors and the general warm home-like environment – ‘We called it the house’ (Hannah). Not only did the environment implicitly communicate safety, but it also allowed participants to communicate and experiment with the expression of their own unique identity through personal dress, interpersonal engagement and engagement in adjunctive therapies (e.g. equine, art, exercise and nature-based therapies).

#### Theme 2b: communication and consistency

As participants discussed communication and consistency in care, tensions emerged in their narratives between individual freedom and a desire for consistency and structure within treatment. Although all participants acknowledged the value of the personalised approach, a number also described how consistency in treatment approaches, including adherence and delivery of treatment non-negotiables^32^ and the quality of care received between staff members, created a sense of uncertainty. For example, although many participants expressed an initial disdain of treatment non-negotiables and aspects of the treatment environment they perceived as restrictive (e.g. limited access to telephones, only being permitted three disliked foods and the busy schedule), they also described the same environment as being instrumental in removing barriers and creating a space that allowed them to engage in treatment.

The weekly multidisciplinary team meetings were an area where inconsistencies in treatment experience were frequently noticed. Some participants found them to be ‘punitive’ and ‘disorganised’ (Bourke), whereas others felt they were walking into a ‘room of cheerleaders’ (Sarah). Several participants felt the feedback provided in the multidisciplinary team meeting was, at times, incongruent with the messaging they had received throughout the week. Numerous individuals voiced a desire to receive more regular feedback from staff throughout the week to help them understand their progress in relation to goals. Participants stressed the need for consistent communication, particularly in relation to treatment non-negotiables, progress and programme implementation, to foster a ‘safe’ environment. Thus, within participant narratives were ideological dilemmas^33^ that were embedded in the divergent philosophies that informed the endeavour to implement replicable standardised phase-based treatment protocols across the collective of participants and individualised and person-centred care.

#### Theme 2c: ‘recovery takes a village’

In speaking to their experience of residential treatment, participants most frequently commented on the value of relationships. A unique aspect of residential treatment for an eating disorder is living in a home-like environment and interacting with others experiencing similar difficulties. For many participants, the milieu and staff functioned as a formative experience that was central to the narrative of their treatment experience.

Participants frequently highlighted differences between their experience of the residential milieu and previous in-patient treatments. Several described residential care as offering community that fostered participant solidarity in their recovery while mitigating unhelpful comparisons that invited competitiveness around eating and body weight/shape. However, participants also described challenging aspects of the milieu, such as proximity to other participants who were at a lower weight and the everchanging dynamic within the milieu as participants entered and left the programme. Many participants also reflected on the pivotal role of the core process group. Offered three times per week, this psychotherapy group enabled participants to share recovery successes and challenges and address dynamics within the milieu, including managing interpersonal challenges and conflicts.

Participants also spoke on the value of the lived experience of others – including peers and lived experience staff members – in providing hope for recovery and snapshots of the many possible versions of recovery. The high level of lived experience personnel at the facility was universally seen as helpful, particularly the unique position of recovery navigator – peer support workers with lived experience of eating disorders who assist participants in navigating the programme by providing daily individual and group support, including meal assistance and engagement in therapeutic activities. Participants described recovery navigators as providing hope with their presence – ‘Seeing that they had existed past the point of having an eating disorder… that blew my mind’ (Lily) – in addition to being a ‘safe’, non-clinical person they could approach for practical advice and support.

### Theme 3: transitions

Participants unanimously emphasised the necessity for enhanced discharge planning and community transition pathways. Discharge from residential treatment was described as ‘very difficult’ (Grace). Many participants reported finding it difficult or being unable to access and/or link-in with sufficient community treatment supports upon returning to their community. This was particularly evident for individuals who were interstate or from rural and remote regions.

Furthermore, all participants reflected on the loss of community and at times feeling ‘homesick’ (Michelle) for the residential treatment community, with several participants attributing the sense of ‘isolation’ (Mia) and lack of community as contributing factors to relapse following discharge. Within this context, participants unanimously expressed the need for continuity of care and collaborative discharge planning throughout the residential treatment journey. They advocated for improved discharge planning and community transition pathways. For example, having a step-down programme with reduced supports, moving to a community day programme on discharge or planned telephone check-ins following discharge.

### Cross-cutting theme: barriers, setbacks and hopes

Treatment barriers were most frequently talked about in relation to financial barriers. Almost all participants spoke of only being able to access the facility through the financial support of their carers/family members, health insurance and/or a bursary. Individuals who left early, due to medical advice, or experienced relapse following discharge often cited finances as being a key barrier to either an extension of their stay – to complete higher phases of care – or considering readmission. Furthermore, interstate participants commented on logistical barriers, such as the location of the facility, being a significant consideration and barrier to access.

Treatment narratives were marked by internal conflict as individuals navigated a sense of liminality in their relationship with themselves and the eating disorder toward a rediscovery of self and identity. Participants consistently acknowledged their experience of residential treatment was shaped by their own motivation, readiness to change and hopes for a life lived differently. Participants who felt ‘forced’ to engage in treatment reported difficulty engaging in the programme. Conversely, those who independently made the decision to seek residential treatment were more likely to embrace the programme and report treatment goals being aligned with their own personal values and goals. Participants who discharged early spoke about the experience in ways that highlighted autonomy. For example, in reflecting on her decision to decline a treatment extension, Enola reflected on the dignity afforded by ‘doing’ treatment ‘on my own terms’, with the opportunity to leave ameliorating the sense of entrapment previously experienced in treatments.

## Discussion

In contrast with people experiencing hospital care as disqualifying their identity,^14,18^ the present study found participants’ perceived residential treatment to be ‘giving identity’. This finding aligns with literature advocating for patient-centred and recovery-oriented care,^34^ emphasising the importance of ‘seeing the person’^21,35^, acknowledging the individual beyond their illness, and considering the roles of identity, relationships and environment.

Drawing on social constructionist frameworks,^30^ this study highlights the potential influence of social and environmental contexts on individual learning and identity within eating disorder treatment. Participant accounts echoed the growing body of literature suggesting that finding one’s identity outside of the eating disorder may be central to recovery,^36^ with participants stressing the value of collaborative care, consistency, communication and community within the residential setting in fostering identity. For many, the treatment milieu was experienced as a recursive source of hope. Participants spoke to finding hope for recovery in witnessing the recovery journeys of peers and having access to staff members with lived experience. These findings align with previous research suggesting the presence of lived-experience staff members and peer support workers may enhance individuals’ sense of hope or optimism for recovery, reduce stigma and improve motivation for treatment engagement.^37^

Building on previous literature,^20,23,24^ this study found that although the core foundations of residential treatment may be similar to those provided in intensive out-patient or in-patient treatment programmes (e.g. psychoeducation, psychological therapy, nutritional rehabilitation, groups), the home-like environment distinguishes this model of care. Additionally, the non-punitive, relationally centred approach to treatment delivery further sets it apart. Moreover, this study underscores the significance of seemingly ‘small things’, such as clothing, presentation of food and facility furnishings, in fostering a sense of normality and safety within the treatment environment. Participants not only perceived the treatment environment as conveying safety, but also appreciated the opportunities it provided for expressing their identity through personal attire and participation in adjunctive therapies. These findings build on Verschueren and colleagues’^38^ assertation that the treatment context and environment may facilitate patients in exploring and discovering their own identity.

Furthermore, similar to in-patient treatment, narratives revealed a tension between individual autonomy and standardised phase-based treatment protocols in a residential setting. Although participants acknowledged the necessity of treatment non-negotiables, they emphasised that the communication and consistency in their application profoundly influenced their treatment experience. As Geller and Srikameswaran^32^ noted, when ‘non-negotiables appear arbitrary, clients may view treatment providers as careless’, undermining confidence in the care team and negatively affecting the therapeutic alliance. Therefore, treatment non-negotiables, in a residential setting, may be best developed in response to the client population, treatment context and organisational values, with reflexive input from participants and clinicians. Integrating constant treatment protocols with individualised care and timely feedback may better support client autonomy while maintaining essential structure.

Residential care offers a unique opportunity to facilitate person-centred and recovery-oriented treatment that promotes self-determination and autonomy in the context of individualised, holistic and evidenced-based person-centred treatment, with participants in this study, for the most part, reporting feeling ‘seen’ and ‘empowered’ to be a part of their own treatment team. However, residential treatment remains only one option along the continuum of care. As such, identifying individuals who may benefit most from this form of treatment is critical given the significant financial and time-related barriers associated with residential care. Furthermore, as with in-patient treatment settings,^12,18^ inherent tensions arise between administering replicable standardised phase-based treatment protocols and participants’ desire for individualised person-centred care (e.g. treatment non-negotiables versus personalised care). Further research is needed to determine how these tensions may be navigated. Additionally, recovery – irrespective of its definition – does not exist in a vacuum.^33,39^ As such, intentionally integrated stepped care models of discharge planning may support individuals in receiving continuity of care and community upon transition to community care.

### Limitations and future directions

A strength of this study was the recruitment from multiple participant cohorts over 2 years of service operation. However, the findings need to be interpreted in the context of participants being recruited from a single service, meaning they were required to meet specific intake criteria, such as medical stability. Additionally, there was limited diversity in eating disorder diagnoses, with two-thirds of participants diagnosed with anorexia nervosa, which reflects the broader demographics of those admitted. Furthermore, individuals who chose to participate may have experienced a heightened level of participant satisfaction or dissatisfaction with the service. Despite these limitations, by drawing on the voices of participants, this study offers new insights into individual lived experiences of residential treatment. Being a qualitative study, findings in this study should not be interpreted as a solution or diagnostic answer but seen as a representation of a set of individuals’ experiences.^40^

Further research is needed to explore participant experiences in other residential services and should include diverse samples to better understand the impact of gender, culture, illness duration and diagnostic factors on treatment experiences. This may provide insight into who may benefit most from this model of care and how residential care may be further tailored to meet the needs of each individual. Additionally, exploring the perspectives of healthcare professionals, lived-experience staff members and caregivers may further enrich current understandings of residential treatment. An analysis of these experiences may play an important role in the future development of the model of care, with a view to improving treatment outcomes.

In conclusion, this study gives voice to the lived experience of those who engage in residential treatment for an eating disorder. Findings highlight the unique opportunity presented by the residential model as well as the inherent ideological dilemmas that arise when balancing replicable standardised phase-based treatment protocols with person-centred and recovery-oriented care. This study found that the residential model offers a unique opportunity to facilitate person-centred and recovery-oriented treatment that promotes self-direction and autonomy in the context of individualised, holistic and evidenced-based, person-centred treatment. However, residential treatment remains only one option along the continuum of care. As such, the importance of identifying individuals who may benefit most from this form of treatment is critical, given the significant financial and time-related barriers associated with residential care.

## Supporting information

Rankin et al. supplementary material 1 Rankin et al. supplementary material

Rankin et al. supplementary material 2 Rankin et al. supplementary material

Rankin et al. supplementary material 3 Rankin et al. supplementary material

## Data Availability

The additional files contain the following data and materials for study: criterion of admission for treatment at time of study (Supplementary File 1), a summary of the lived experience advisory group (Supplementary File 2) and author reflexivity statements (Supplementary File 3). Exemplar data extracts for the themes and meta themes are presented in the findings (Table 1). In line with the ethics approvals for this study, original data that support the findings of this study are not publicly available as they contain information that could compromise the privacy and confidentiality of research participants.

## Supplementary material

The supplementary material is available online at https://doi.org/10.1192/bjo.2024.849
