# Supplementary material for: ‘It takes a village’: patient lived experiences of residential treatment for an eating disorder
Source: BJPsych Open. 2025 Feb 3;11(2):e30. doi: 10.1192/bjo.2024.849 (PMC11822953; doi:10.1192/bjo.2024.849)
Supplement: Rankin et al. supplementary material 1 — Rankin et al. supplementary material [file S2056472424008494sup001.docx]

## Additional File 1

**Criteria for admission at time of study^1^**

The service is open to individuals of any gender aged 16 years or older, with a primary diagnosis of an eating disorder under the DSM-5. Other admission criteria include the following: 1) BMI of >13.5; 2) absence of any significant self-harm or suicidal ideation; 3) being willing and able to consent to treatment; 4) independent management of mobility; 5) no active substance dependence; 6) manageable dietary restrictions (e.g. no veganism but most religious and/or cultural dietary considerations can be catered for and this will be discussed during your intake assessment), and medical stability as evidenced by a systolic BP 90mm (>80mm for adolescents) or above, heart rate >50 and <100 bpm, no significant postural tachycardia or hypotension, no acute ECG changes and, electrolytes managed by oral treatment. Anaphylactic allergies were considered on a case-by-case basis following diagnostic testing and consultation with a treating primary healthcare provider or physician.

Individuals may be referred to the service by their treating primary health care provider or psychiatrist. Upon referral, individuals are screened for eligibility and participate in an intake assessment facilitated by a senior team member of the team (e.g., Clinical Director or Clinical Lead, Nurse Unit Manager) as it must be determined that residential treatment is the most appropriate approach available on the continuum of care for the individual at that given point in time.

## References

1. Wandi Nerida. For referrers: admission criterion. The Butterfly Foundation, 2022(https://wandinerida.hothealth.com/for-referrers [cited 15 Jun 2022])
