# Supplementary material for: ‘It takes a village’: patient lived experiences of residential treatment for an eating disorder
Source: BJPsych Open. 2025 Feb 3;11(2):e30. doi: 10.1192/bjo.2024.849 (PMC11822953; doi:10.1192/bjo.2024.849)
Supplement: Rankin et al. supplementary material 2 — Rankin et al. supplementary material [file S2056472424008494sup002.docx]

## Additional File 2

**Lived experience advisory group**

A lived experience advisory panel, consisting of five lived experience individuals (three lived experience individuals; one lived experience parent/carer; one lived experience health care practitioner) was developed prior to the commencement of this study. This panel provided feedback during the design of participant interview schedules and participant materials. This feedback was incorporated into the final interview schedule and participant information sheets that were used in this study. This consultation was done to ensure that the participant information and consent materials were easy to understand. Additionally, seeking feedback also identified where the researchers may not have thought to ask about specific aspects of the participant experience. Literature and anecdotal evidence indicate that consultation with individuals who belong to and/or represent the interests of those with eating disorders may aid the researchers in conducting research that is informed by real-world knowledge and experiences which has the potential to translate into relevant and meaningful research findings.^1,2,3^ The researchers wish to acknowledge the valuable contribution of the lived experience advisory group.

**References**

1. Güell E, Benito-Amat C, Molas-Gallart J. Priority setting in mental health research: a scoping review of participatory methods. *Ment Health Prev.* 2023; 30:200279.

2. Duea SR, Zimmerman EB, Vaughn LM, Dias S, Harris J. A guide to selecting participatory research methods based on project and partnership goals. *J Participle Res Methods*. 2022; 3(1):10.35844/001c.32605

3. Byrne L, Wang Y, Roennfeldt H, Chapman M, Darwin L, Craze L, Saunders M. National Mental Health Commission. National Lived Experience (Peer) Workforce Development Guidelines: Placing lived experience at the center of mental health reform. National Mental Health Commission, 2021 (https://www.mentalhealthcommission.gov.au/publications/national-lived-experience-peer-workforce-development-guidelines [cited 1 Jun 2024])
