# Supplementary material for: ‘It takes a village’: patient lived experiences of residential treatment for an eating disorder
Source: BJPsych Open. 2025 Feb 3;11(2):e30. doi: 10.1192/bjo.2024.849 (PMC11822953; doi:10.1192/bjo.2024.849)
Supplement: Rankin et al. supplementary material 3 — Rankin et al. supplementary material [file S2056472424008494sup003.docx]

**Additional File 3**

**Author Reflexivity Statements**

In adopting reflexive thematic analysis within a social constructionist perspective, the authors acknowledge the influence of researcher positionality on the processes of data generation, analysis, and presentation. Consequently, the authors feel it is imperative that they position themselves in relation to this analytical framework. All authors identify as female, cisgender clinician researchers based in Sydney, Australia. Collectively, the authors possess expertise in both qualitative and quantitative research methods, as well as in the field of eating disorders and their treatment, drawing from a combination of lived experiences, clinical practice, and educational training. This breadth of experience provides the authors with a unique perspective through which they interpret the data. While the authors diverse backgrounds enable them to attune to various aspects of the data, the authors acknowledge the possibility of analytical gaps resulting from their shared positionality. Individual positioning statements are provided below.

### Rebekah Rankin

I am an Anglo-European female, doctoral student and clinical psychologist, with a lived experience of disordered eating and participation in competitive sport. As a clinician I work in private practice and have a special interest in providing evidence-based interventions to those who experience difficult relationships with their bodies, food, eating and/or exercise. As a researcher my experience pertains primarily to conducting research in the areas of motivation, disordered eating, and exercise dependence, across both qualitative and quantitively methodologies. While my own personal history does not include any inpatient or residential treatment experiences, I acknowledge that researchers—qualitative and quantitative alike—are never neutral observers in their positioning and bring with them their own knowledge, theoretical assumptions and curiosities about any given phenomena.

As a researcher I believe that qualitative research is about meaning and the process of meaning-making. I acknowledge that these meanings are contextually bound, positioned and situated. Building the insights of Braun and Clarke,^1,2^ I contend that themes do not passively emerge from either data or coding but rather; instead, they represent interpretive narratives crafted from the data, emerging at the confluence of the researcher's theoretical perspectives, analytical capabilities, and the data itself. Consequently, the analyses and findings presented in this study stem from extensive immersion in the data, accompanied by deep reflection on the assumptions underlying our roles as both researchers and clinicians. It is important to acknowledge the ongoing nature of the reflexive process of analysis, as such analysis can never be considered fully complete.^3,4^

The current dataset presented us with a rich tapestry of complex and intricate data, replete with nuanced narratives and a multitude of interpretive possibilities. The primary challenge in this research was the act of choosing which stories to tell given the limitations of the current publication word count. Hence, guided by feedback from our participants, consultations with a lived experience advisory group, and discussions within our broader clinical evaluation team, I posit that the analysis offered here represents one of numerous possible authentic interpretations of the data. To bolster the analytical rigor and integrity of this work, I engaged in regular external supervision to capture/process personal responses to the research process and findings. Additionally, fortnightly meetings were also held between myself and my co-authors to ensure methodological rigour and discuss the data and analytical ‘noticing’s’.

### Janet Conti

I am a Clinical Psychologist, Dietitian and academic in Clinical Psychology. My research and clinical work seek to prioritise the voice of the experiencing person to inform the development of a greater number of effective treatment interventions for AN that are tailored to the needs and preferences of the experiencing person and their family.

### Lucie Ramjan

I am a Registered Nurse and Professor of Nursing. My research expertise is qualitative and has centred on learning more about the personal experiences of treatment and recovery for people with eating disorders. This research supports finding adjuncts to treatment that empower and inspire hope for recovery.

### Phillipa Hay

I am an academic Psychiatrist with long-standing clinical and research experience in the treatment of people with anorexia nervosa. I am very interested to explore and understand better how to improve treatments, reduce distress during treatment, and in particular to better understand why some people have poor outcomes.

## References

1. Braun V, Clarke V, Hayfield N, Terry G. Thematic Analysis. In *Handbook of research methods in health social sciences* (ed Liamputtong P); 843–60. Springer; 2019.
2. Braun V, Clarke V. Toward good practice in thematic analysis: Avoiding common problems and be(com)ing a *knowing* researcher. *Int J Transgender Health.* 2023; 24(1):1–6.
3. Ho KHM, Chiang VCL, Leung D. Hermeneutic phenomenological analysis: the ‘possibility’ beyond ‘actuality’ in thematic analysis. *J Adv Nurs.* 2017; 73(7):1757–66.
4. Low J. A pragmatic definition of the concept of theoretical saturation. *Sociological focus.* 2019;v52(2):131-9.
